# Supplementary material for: The relationship between emotional disorders and heart rate variability: A Mendelian randomization study
Source: PLoS One. 2024 Mar 7;19(3):e0298998. doi: 10.1371/journal.pone.0298998 (PMC10919610; doi:10.1371/journal.pone.0298998)
Supplement: S1 Table — (DOCX) [file pone.0298998.s001.docx]

**S1Table. Specific information on instrumental variables for seven types of emotional disorders**

| **SNP** | **Chr** | **Position** | **EA** | **OA** | **EAF** | **BEAT** | **SE** | **P** | **F-statistic** | **R2** |
| --- | --- | --- | --- | --- | --- | --- | --- | --- | --- | --- |
| **Depression (broad)** |  |  |  |  |  |  |  |  |  |  |
| **rs1021363** | 10 | 106610839 | G | A | 0.642 | -0.007 | 0.001 | 1.04E-08 | 1837.932 | 0.006 |
| **rs10501696** | 11 | 88748162 | G | A | 0.499 | -0.008 | 0.001 | 6.73E-11 | 2287.935 | 0.007 |
| **rs2402273** | 7 | 117600424 | C | T | 0.409 | 0.007 | 0.001 | 1.95E-09 | 1933.527 | 0.006 |
| **rs2568958** | 1 | 72765116 | A | G | 0.600 | 0.009 | 0.001 | 4.81E-13 | 2472.383 | 0.008 |
| **rs263575** | 9 | 17033840 | A | G | 0.460 | -0.007 | 0.001 | 2.31E-08 | 1987.298 | 0.006 |
| **rs3132685** | 6 | 29945949 | A | G | 0.130 | -0.013 | 0.002 | 2.47E-13 | 837.236 | 0.003 |
| **rs3807865** | 7 | 12250402 | A | G | 0.412 | 0.008 | 0.001 | 7.28E-12 | 2216.586 | 0.007 |
| **rs7548151** | 1 | 177026983 | A | G | 0.084 | 0.013 | 0.002 | 3.87E-09 | 569.115 | 0.002 |
| **Major Depressive Disorder** |  |  |  |  |  |  |  |  |  |  |
| **rs1343607** | 13 | 53645448 | G | A | 0.618 | -0.074 | 0.015 | 1.00E-06 | 715.624 | 0.008 |
| **rs1504721** | 11 | 99268617 | C | A | 0.390 | 0.070 | 0.015 | 3.98E-06 | 681.926 | 0.007 |
| **rs1676905** | 7 | 148671277 | G | A | 0.925 | 0.070 | 0.015 | 3.36E-06 | 197.831 | 0.002 |
| **rs2414116** | 15 | 52017345 | G | A | 0.547 | -0.070 | 0.015 | 3.43E-06 | 710.495 | 0.008 |
| **rs3131115** | 6 | 30468791 | T | C | 0.370 | -0.081 | 0.015 | 1.12E-07 | 773.925 | 0.008 |
| **rs3807866** | 7 | 12250378 | A | G | 0.403 | 0.081 | 0.015 | 1.04E-07 | 799.009 | 0.009 |
| **Obsessive Compulsive Disorder** |  |  |  |  |  |  |  |  |  |  |
| **rs12568997** | 1 | 88867185 | A | G | / | -0.2933 | 0.058 | 4.23E-07 | 25.57 | / |
| **rs4733767** | 8 | 128581578 | A | G | / | 0.193501 | 0.039 | 7.10E-07 | 24.62 | / |
| **rs639560** | 15 | 42381240 | T | C | / | -0.4158 | 0.0879 | 2.23E-06 | 22.38 | / |
| **rs9952159** | 18 | 3660801 | T | C | / | 0.182297 | 0.0396 | 4.21E-06 | 21.19 | / |
| **Bipolar Disorder** |  |  |  |  |  |  |  |  |  |  |
| **rs10744560** | 12 | 2387099 | T | C | 0.342 | 0.083 | 0.014 | 2.92E-09 | 613.944 | 0.012 |
| **rs17150022** | 7 | 24771777 | C | T | 0.121 | 0.113 | 0.020 | 2.70E-08 | 274.742 | 0.005 |
| **rs2071044** | 3 | 52847601 | T | C | 0.468 | -0.078 | 0.014 | 9.09E-09 | 638.639 | 0.012 |
| **rs329319** | 5 | 133906609 | G | A | 0.566 | -0.079 | 0.014 | 1.54E-08 | 638.165 | 0.012 |
| **rs9834970** | 3 | 36856030 | C | T | 0.501 | 0.101 | 0.013 | 5.53E-14 | 898.669 | 0.017 |
| **Irritable Mood** |  |  |  |  |  |  |  |  |  |  |
| **rs102275** | 11 | 61557803 | T | C | 0.644 | -0.014 | 0.002 | 1.30E-08 | 1954.071 | 0.005 |
| **rs11682175** | 2 | 57987593 | T | C | 0.534 | -0.013 | 0.002 | 1.20E-08 | 1969.542 | 0.005 |
| **rs12886000** | 14 | 98501877 | T | G | 0.106 | 0.021 | 0.004 | 3.33E-08 | 603.553 | 0.002 |
| **rs13223152** | 7 | 69948241 | A | G | 0.598 | 0.015 | 0.002 | 1.14E-09 | 2196.750 | 0.006 |
| **rs1542212** | 3 | 35683935 | T | G | 0.607 | -0.014 | 0.002 | 1.93E-08 | 2033.665 | 0.006 |
| **rs2217127** | 18 | 35221630 | T | G | 0.291 | 0.014 | 0.003 | 3.95E-08 | 1169.843 | 0.003 |
| **rs2587410** | 18 | 63536017 | T | C | 0.747 | -0.015 | 0.003 | 1.05E-08 | 1148.067 | 0.003 |
| **rs3026401** | 11 | 31807524 | T | C | 0.782 | -0.017 | 0.003 | 1.41E-09 | 1173.754 | 0.003 |
| **rs3124405** | 13 | 55970997 | T | G | 0.723 | -0.017 | 0.003 | 9.48E-11 | 1379.669 | 0.004 |
| **rs3772556** | 3 | 105249211 | T | C | 0.712 | -0.014 | 0.003 | 2.26E-08 | 1162.659 | 0.003 |
| **rs3774800** | 3 | 49334768 | A | G | 0.646 | -0.015 | 0.002 | 1.40E-09 | 2089.116 | 0.006 |
| **rs4411173** | 1 | 98507718 | A | C | 0.837 | 0.019 | 0.003 | 5.07E-09 | 1049.506 | 0.003 |
| **rs4734804** | 8 | 105696848 | A | G | 0.780 | 0.016 | 0.003 | 3.51E-08 | 1111.806 | 0.003 |
| **rs7231748** | 18 | 53109035 | A | G | 0.693 | -0.017 | 0.003 | 6.51E-12 | 1465.988 | 0.004 |
| **rs7535528** | 1 | 2444414 | A | G | 0.363 | 0.014 | 0.002 | 1.93E-08 | 1970.926 | 0.005 |
| **rs9938550** | 16 | 30999142 | A | G | 0.385 | -0.014 | 0.002 | 6.21E-09 | 2018.441 | 0.005 |
| **Anxiety Disorder** |  |  |  |  |  |  |  |  |  |  |
| **rs10493298** | 1 | 61336529 | T | C | / | -0.004 | 0.001 | 1.18E-06 | 23.61 | / |
| **rs1333904** | 9 | 122674412 | C | T | / | -0.005 | 0.001 | 6.61E-09 | 33.65 | / |
| **rs2254382** | 20 | 37379726 | C | A | / | -0.004 | 0.001 | 4.58E-06 | 21.00 | / |
| **rs2272287** | 7 | 2653792 | A | C | / | 0.004 | 0.001 | 1.83E-06 | 22.77 | / |
| **rs2514218** | 11 | 113392994 | T | C | / | -0.004 | 0.001 | 1.67E-06 | 22.94 | / |
| **rs4869077** | 5 | 167259572 | A | G | / | 0.007 | 0.001 | 3.47E-06 | 21.54 | / |
| **rs542852** | 5 | 78409396 | T | C | / | 0.005 | 0.001 | 1.92E-07 | 27.11 | / |
| **rs6460894** | 7 | 12247330 | C | T | / | 0.004 | 0.001 | 2.96E-06 | 21.84 | / |
| **rs719350** | 2 | 155913473 | G | A | / | -0.005 | 0.001 | 3.86E-06 | 21.33 | / |
| **rs7506245** | 18 | 50570403 | G | A | / | 0.004 | 0.001 | 3.15E-07 | 26.16 | / |
| **rs847503** | 14 | 36471214 | A | G | / | 0.009 | 0.002 | 2.03E-06 | 22.57 | / |
| **rs9276931** | 6 | 32928984 | G | A | / | -0.007 | 0.001 | 1.72E-07 | 27.33 | / |
| **rs942866** | 14 | 104014935 | G | T | / | -0.004 | 0.001 | 1.16E-06 | 23.65 | / |
| **Mania** |  |  |  |  |  |  |  |  |  |  |
| **rs11130252** | 3 | 50591727 | A | G | 0.124 | 0.114 | 0.024 | 2.34E-06 | 396.490 | 0.003 |
| **rs11720844** | 3 | 142556399 | G | A | 0.228 | -0.101 | 0.019 | 1.04E-07 | 720.589 | 0.005 |
| **rs17753324** | 7 | 42179545 | T | G | 0.187 | -0.102 | 0.021 | 7.59E-07 | 568.109 | 0.004 |
| **rs2506153** | 10 | 33484922 | A | G | 0.103 | 0.125 | 0.026 | 2.38E-06 | 341.206 | 0.002 |
| **rs6974707** | 7 | 55982894 | A | G | 0.223 | 0.089 | 0.019 | 3.41E-06 | 624.666 | 0.004 |
