# Supplementary material for: The relationship between emotional disorders and heart rate variability: A Mendelian randomization study
Source: PLoS One. 2024 Mar 7;19(3):e0298998. doi: 10.1371/journal.pone.0298998 (PMC10919610; doi:10.1371/journal.pone.0298998)
Supplement: S2 Table — (DOCX) [file pone.0298998.s002.docx]

**S2Table. Heterogeneity and sensitivity analysis results for emotional disorders and HRV (pvRSA/HF)**

| **Outcome** | **Exposure** | **Inverse variance weighted** |  | **MR Egger** |  | **Egger** | **MR-PRESSO results Global Test** |  |  |
| --- | --- | --- | --- | --- | --- | --- | --- | --- | --- |
|  |  | Q-statistic | P | Q-statistic | P | P | RSSobs | P | Outliers |
|  | Depression (broad) | 6.959 | 0.433 | 6.868 | 0.333 | 0.787 | 9.076 | 0.447 | No |
|  | Major Depressive Disorder | 0.159 | 0.923 | 0.073 | 0.787 | 0.059 | 15.060 | 0.090 | No |
| Heart rate variability traits (pvRSA/HF) | Obsessive Compulsive Disorder | 5.714 | 0.126 | 2.860 | 0.239 | 0.293 | 9.872 | 0.201 | No |
|  | Bipolar Disorder | 5.659 | 0.226 | 5.626 | 0.131 | 0.903 | 10.031 | 0.254 | No |
|  | Irritable Mood | 23.823 | 0.068 | 23.737 | 0.049 | 0.826 | 26.923 | 0.079 | No |
|  | Anxiety Disorder | 11.242 | 0.508 | 11.127 | 0.433 | 0.742 | 13.016 | 0.530 | No |
|  | Mania | 2.391 | 0.664 | 1.155 | 0.764 | 0.347 | 3.963 | 0.662 | No |
