# Supplementary material for: The relationship between emotional disorders and heart rate variability: A Mendelian randomization study
Source: PLoS One. 2024 Mar 7;19(3):e0298998. doi: 10.1371/journal.pone.0298998 (PMC10919610; doi:10.1371/journal.pone.0298998)
Supplement: S3 Table — (DOCX) [file pone.0298998.s003.docx]

**S3 Table. Heterogeneity and sensitivity analysis results for emotional disorders and HRV (RMSSD)**

| **Outcome** | **Exposure** | **Inverse variance weighted** |  | **MR Egger** |  | **Egger** | **MR-PRESSO results Global Test** |  |  |
| --- | --- | --- | --- | --- | --- | --- | --- | --- | --- |
|  |  | Q-statistic | P | Q-statistic | P | P | RSSobs | P | Outliers |
|  | Depression (broad) | 8.433 | 0.296 | 8.403 | 0.210 | 0.888 | 10.743 | 0.337 | No |
|  | Major Depressive Disorder | 4.540 | 0.474 | 1.279 | 0.865 | 0.145 | 6.776 | 0.484 | No |
| Heart rate variability traits (RMSSD) | Obsessive Compulsive Disorder | 1.144 | 0.766 | 0.840 | 0.657 | 0.637 | 2.367 | 0.745 | No |
|  | Bipolar Disorder | 0.294 | 0.990 | 0.245 | 0.970 | 0.838 | 0.571 | 0.984 | No |
|  | Irritable Mood | 19.890 | 0.176 | 19.888 | 0.134 | 0.976 | 22.563 | 0.192 | No |
|  | Anxiety Disorder | 8.820 | 0.718 | 7.992 | 0.714 | 0.382 | 9.863 | 0.769 | No |
|  | Mania | 1.277 | 0.865 | 1.259 | 0.739 | 0.903 | 1.761 | 0.905 | No |
