# Supplementary material for: The relationship between emotional disorders and heart rate variability: A Mendelian randomization study
Source: PLoS One. 2024 Mar 7;19(3):e0298998. doi: 10.1371/journal.pone.0298998 (PMC10919610; doi:10.1371/journal.pone.0298998)
Supplement: S4 Table — (DOCX) [file pone.0298998.s004.docx]

**S4 Table.** **Heterogeneity and sensitivity analysis results for emotional disorders and HRV (SDNN)**

| **Outcome** | **Exposure** | **Inverse variance weighted** |  | **MR Egger** |  | **Egger** | **MR-PRESSO results Global Test** |  |  |
| --- | --- | --- | --- | --- | --- | --- | --- | --- | --- |
|  |  | Q-statistic | P | Q-statistic | P | P | RSSobs | P | Outliers |
|  | Depression (broad) | 5.263 | 0.628 | 5.053 | 0.537 | 0.663 | 6.829 | 0.633 | No |
|  | Major Depressive Disorder | 9.390 | 0.094 | 2.146 | 0.709 | 0.055 | 14.008 | 0.126 | No |
| Heart rate variability traits (SNDD) | Obsessive Compulsive Disorder | 1.838 | 0.607 | 1.541 | 0.463 | 0.640 | 3.737 | 0.606 | No |
|  | Bipolar Disorder | 2.069 | 0.723 | 2.014 | 0.569 | 0.831 | 3.105 | 0.737 | No |
|  | Irritable Mood | 17.500 | 0.290 | 17.499 | 0.231 | 0.978 | 19.883 | 0.309 | No |
|  | Anxiety Disorder | 7.997 | 0.785 | 7.379 | 0.768 | 0.448 | 9.579 | 0.772 | No |
|  | Mania | 2.430 | 0.657 | 2.415 | 0.491 | 0.911 | 4.013 | 0.658 | No |
