# Supplementary material for: The relationship between emotional disorders and heart rate variability: A Mendelian randomization study
Source: PLoS One. 2024 Mar 7;19(3):e0298998. doi: 10.1371/journal.pone.0298998 (PMC10919610; doi:10.1371/journal.pone.0298998)
Supplement: S1 Fig — A. Depression.(broad) B. Major Depressive Disorder C. Obsessive Compulsive Disorder D. Bipolar Disorder E. Irritable Mood F. Anxiety Disorder G. Mania. (DOCX) [file pone.0298998.s005.docx]

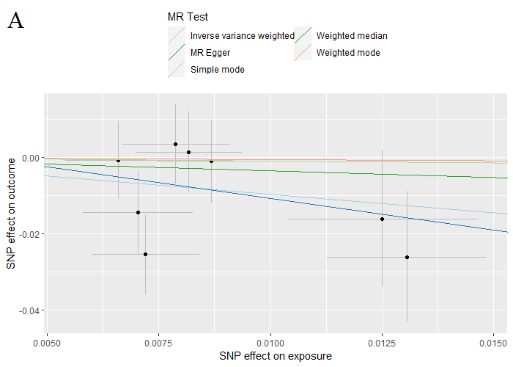

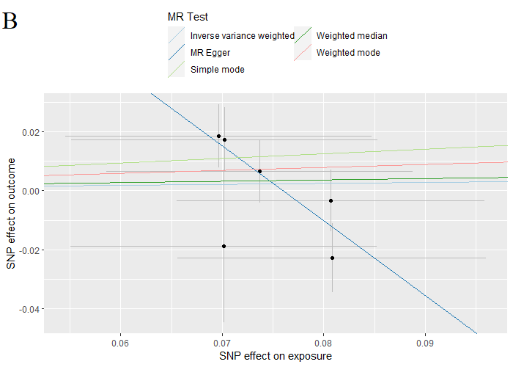

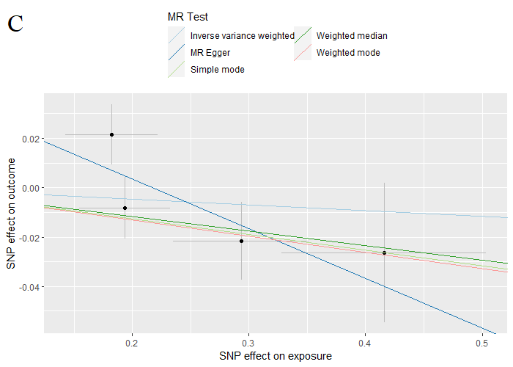

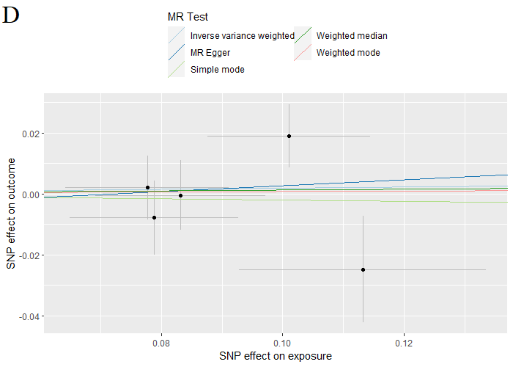

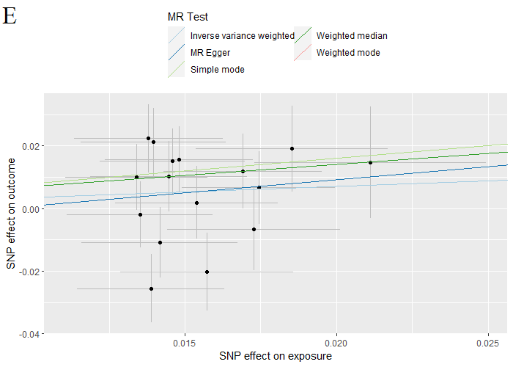

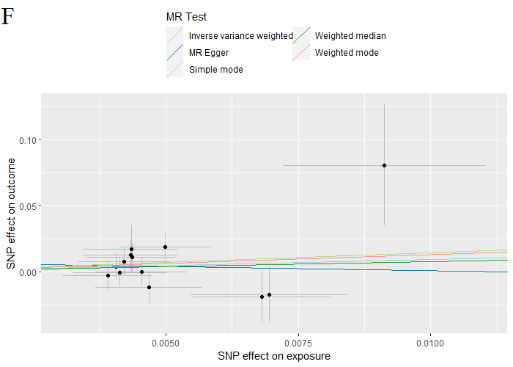

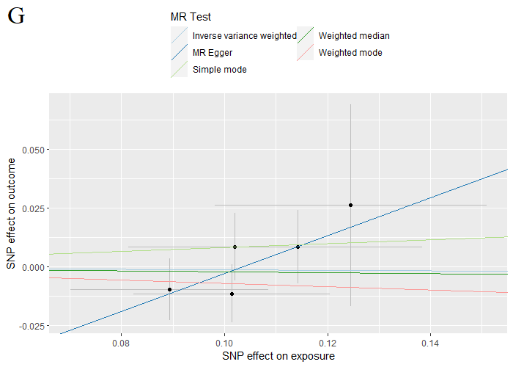


**S1Fig. Scatter plot of heart rate variability (pvRSA/HF) and emotional disorders.** A. Depression.(broad) B. Major Depressive Disorder C. Obsessive Compulsive Disorder D. Bipolar Disorder E. Irritable Mood F. Anxiety Disorder G. Mania
