# Supplementary material for: The relationship between emotional disorders and heart rate variability: A Mendelian randomization study
Source: PLoS One. 2024 Mar 7;19(3):e0298998. doi: 10.1371/journal.pone.0298998 (PMC10919610; doi:10.1371/journal.pone.0298998)
Supplement: S3 Fig — A. Depression(broad) B. Major Depressive Disorder C. Obsessive Compulsive Disorder D. Bipolar Disorder E. Irritable Mood. F. Anxiety Disorder G. Mania. (DOCX) [file pone.0298998.s007.docx]

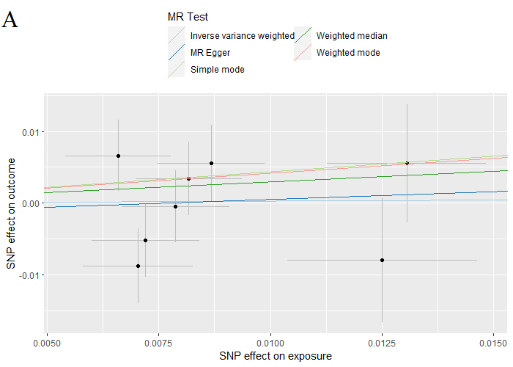

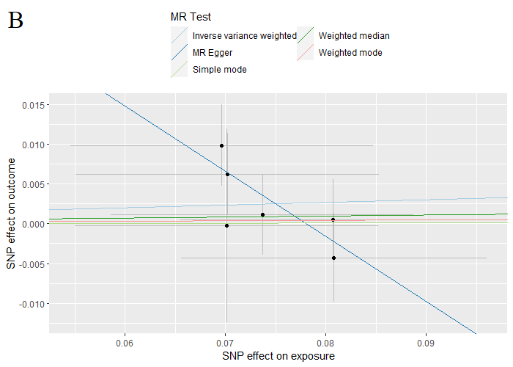

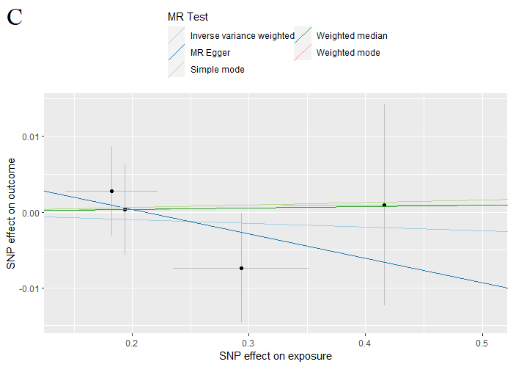

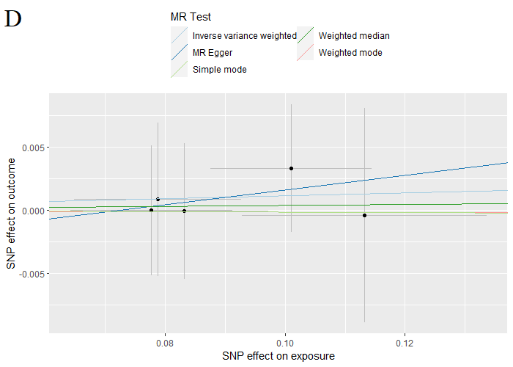

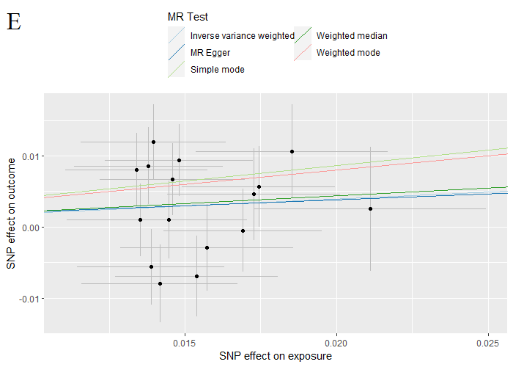

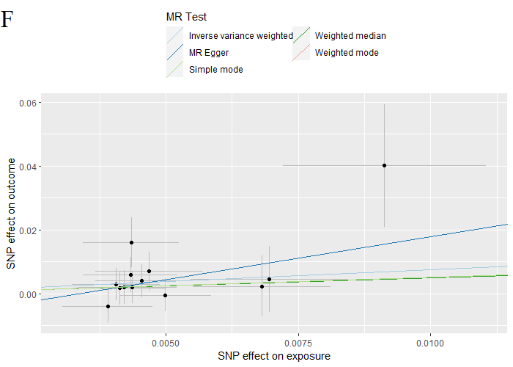


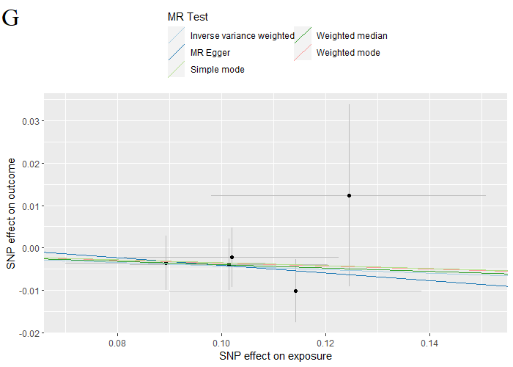


**S3 Fig. Scatter plot of heart rate variability (RMSSD) and emotional disorders.** A. Depression(broad) B. Major Depressive Disorder C. Obsessive Compulsive Disorder D. Bipolar Disorder E. Irritable Mood. F. Anxiety Disorder G. Mania
