# Supplementary material for: The relationship between emotional disorders and heart rate variability: A Mendelian randomization study
Source: PLoS One. 2024 Mar 7;19(3):e0298998. doi: 10.1371/journal.pone.0298998 (PMC10919610; doi:10.1371/journal.pone.0298998)
Supplement: S4 Fig — A. Depression (broad). B. Major Depressive Disorder C. Obsessive Compulsive Disorder D. Bipolar Disorder. E. Irritable Mood F. Anxiety Disorder G. Mania. (DOCX) [file pone.0298998.s008.docx]

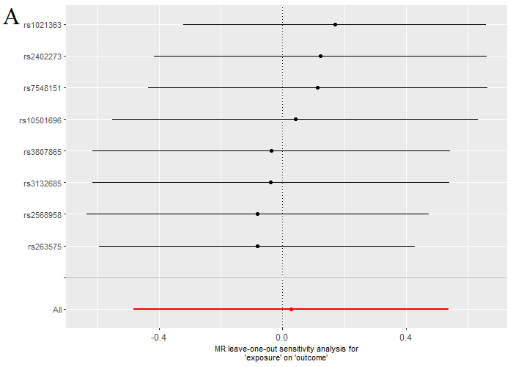

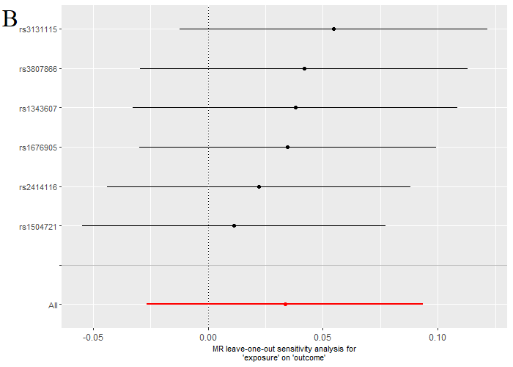

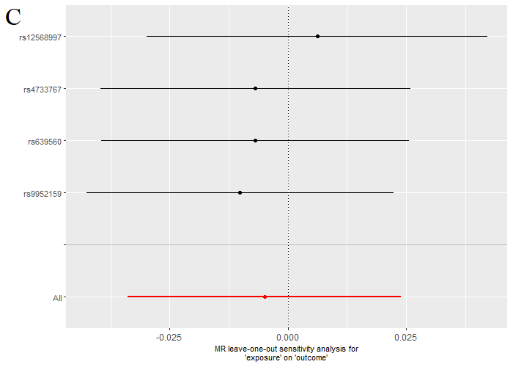

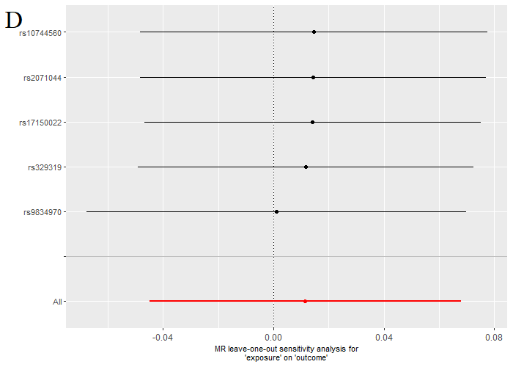

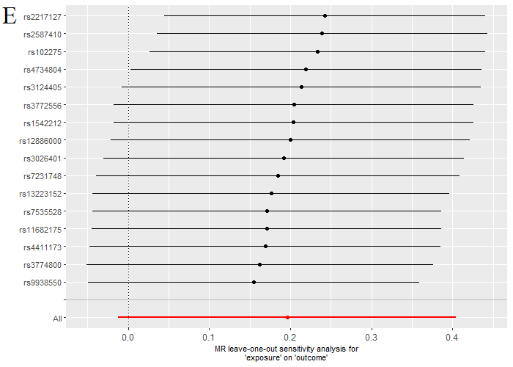

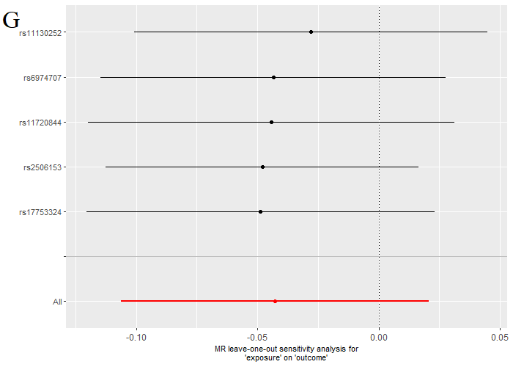

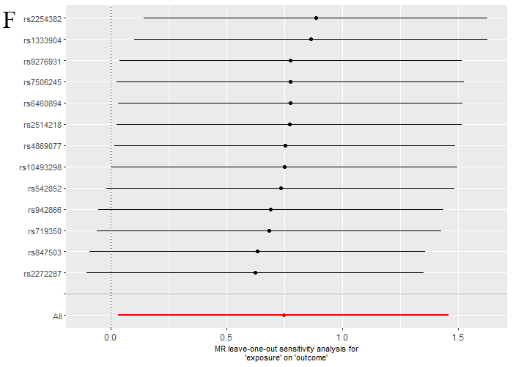


**S4 Fig. Leave-one-out analysis of heart rate variability traits (RMSSD) and emotional disorders.**

A.Depression (broad). B. Major Depressive Disorder C. Obsessive Compulsive Disorder D. Bipolar Disorder.E. Irritable Mood F. Anxiety Disorder G. Mania
