# Supplementary material for: The relationship between emotional disorders and heart rate variability: A Mendelian randomization study
Source: PLoS One. 2024 Mar 7;19(3):e0298998. doi: 10.1371/journal.pone.0298998 (PMC10919610; doi:10.1371/journal.pone.0298998)
Supplement: S6 Fig — A. Depression (broad) B. Major Depressive Disorder C. Obsessive Compulsive Disorder D. Bipolar Disorder. E. Irritable Mood F. Anxiety Disorder G. Mania. (DOCX) [file pone.0298998.s010.docx]

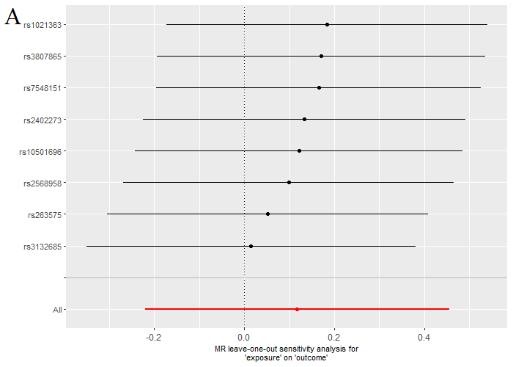

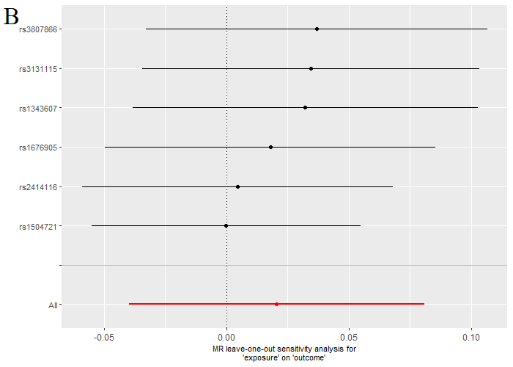

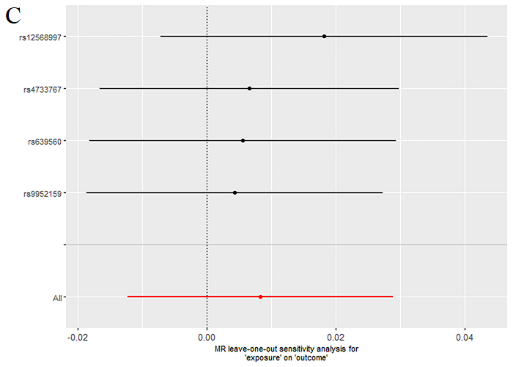

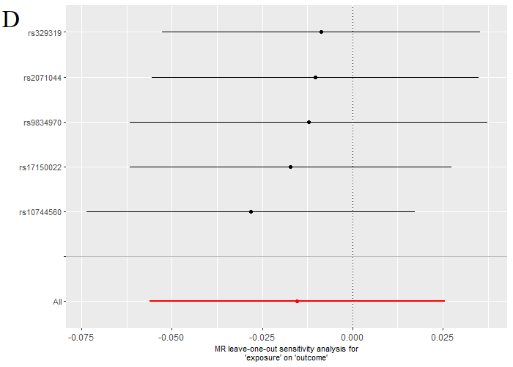

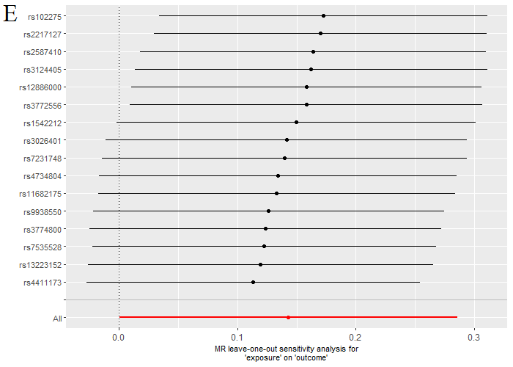

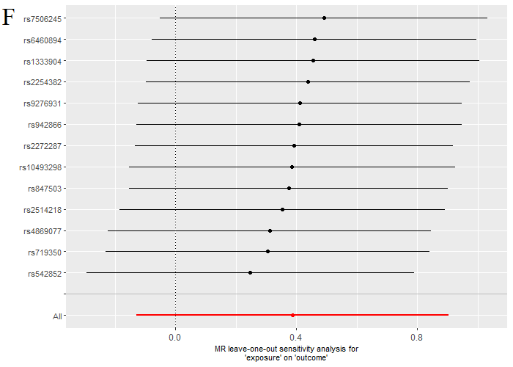

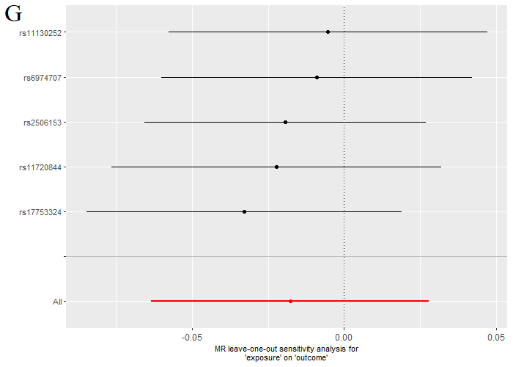


**S6 Fig. Leave-one-out analysis of heart rate variability (SNDD) and emotional disorders.** A.Depression (broad) B. Major Depressive Disorder C. Obsessive Compulsive Disorder D. Bipolar Disorder. E. Irritable Mood F. Anxiety Disorder G. Mania
