# Supplementary material for: The relationship between emotional disorders and heart rate variability: A Mendelian randomization study
Source: PLoS One. 2024 Mar 7;19(3):e0298998. doi: 10.1371/journal.pone.0298998 (PMC10919610; doi:10.1371/journal.pone.0298998)
Supplement: Attachment — Submitted filename: Response to Reviewers.docx [file pone.0298998.s012.docx]

**Reviewer 1**

Thank you very much again for your valuable comments to improve the quality of our manuscript. All questions have been answered in itemized descriptions as shown below.

**Major comments**

**1.**All comments have been addressed, but there are still many typos, grammatical errors and punctuation errors. Despite the authors stated that the manuscript already underwent proofreading, I strongly suggest having it edited once more, referring to a professional native speaker proofreader. The inaccuracies present within the manuscript as of now negatively impact on its quality, and need to be addressed before publication.

**Response:**

Many thanks for your valuable comments for improving the quality of our manuscript. Accordingly, the correction has been made in the Revised Manuscript with Track Changes and Manuscript.

**Reviewer 2:** I thank the authors for their response. I believe they addressed all of my comments satisfactorily. I have no additional comments to add.

Thank you very much again for your valuable comments to improve the quality of our manuscript.
